# Tomato Yield Heterosis Is Triggered by a Dosage Sensitivity of the Florigen Pathway That Fine-Tunes Shoot Architecture

**DOI:** 10.1371/journal.pgen.1004043

**Published:** 2013-12-26

**Authors:** Ke Jiang, Katie L. Liberatore, Soon Ju Park, John P. Alvarez, Zachary B. Lippman

**Affiliations:** 1 Watson School of Biological Sciences, Cold Spring Harbor Laboratory, Cold Spring Harbor, New York, United States of America; 2 Monash University, School of Biological Sciences, Clayton Campus, Melbourne, Victoria, Australia; University of Minnesota, United States of America

## Abstract

The superiority of hybrids has long been exploited in agriculture, and although many models explaining “heterosis” have been put forth, direct empirical support is limited. Particularly elusive have been cases of heterozygosity for single gene mutations causing heterosis under a genetic model known as overdominance. In tomato (*Solanum lycopersicum*), plants carrying mutations in *SINGLE FLOWER TRUSS* (*SFT*) encoding the flowering hormone florigen are severely delayed in flowering, become extremely large, and produce few flowers and fruits, but when heterozygous, yields are dramatically increased. Curiously, this overdominance is evident only in the background of “determinate” plants, in which the continuous production of side shoots and inflorescences gradually halts due to a defect in the flowering repressor *SELF PRUNING* (*SP*). How *sp* facilitates *sft* overdominance is unclear, but is thought to relate to the opposing functions these genes have on flowering time and shoot architecture. We show that *sft* mutant heterozygosity (*sft/+*) causes weak semi-dominant delays in flowering of both primary and side shoots. Using transcriptome sequencing of shoot meristems, we demonstrate that this delay begins before seedling meristems become reproductive, followed by delays in subsequent side shoot meristems that, in turn, postpone the arrest of shoot and inflorescence production. Reducing *SFT* levels in *sp* plants by artificial microRNAs recapitulates the dose-dependent modification of shoot and inflorescence production of *sft/+* heterozygotes, confirming that fine-tuning levels of functional *SFT* transcripts provides a foundation for higher yields. Finally, we show that although flowering delays by florigen mutant heterozygosity are conserved in *Arabidopsis*, increased yield is not, likely because cyclical flowering is absent. We suggest *sft* heterozygosity triggers a yield improvement by optimizing plant architecture via its dosage response in the florigen pathway. Exploiting dosage sensitivity of florigen and its family members therefore provides a path to enhance productivity in other crops, but species-specific tuning will be required.

## Author Summary

For over a century, it has been known that inbreeding harms plant and animal fitness, whereas interbreeding between genetically distinct individuals can lead to more robust offspring in a phenomenon known as hybrid vigor, or heterosis. While heterosis has been harnessed to boost agricultural productivity, its causes are not understood. Especially controversial is a model called “overdominance,” which states in its simplest form that a single gene can drive heterosis, although multiple overdominant genes can also contribute. In tomato, a mutation in just one of two copies of a gene encoding the flowering hormone called florigen causes remarkable increases in yield, but it is not known why. We show that yield increases are triggered by a fine-tuning of florigen levels that cause subtle delays in the time it takes all shoots to produce flowers. The resulting plant architecture maximizes yield in varieties that dominate the processing tomato industry. We show that while similar changes in flowering occur when one copy of florigen is mutated in the model crucifer plant *Arabidopsis*, yield is not increased, suggesting that, while manipulating florigen holds potential to improve crop productivity, the tuning of florigen and related genes will have to be tailored according to species.

## Introduction

More than a century ago, simple garden studies by Darwin revealed a remarkable phenomenon in which crossing related varieties of plants produced hybrid progeny with superior growth and fecundity compared to their parents [1]. Understanding this hybrid vigor began with population genetics theories postulating that outcrossing facilitates adaptation and improves fitness by shuffling allelic diversity to thwart inbreeding depression [2]. However, it was the agricultural exploitation of hybrid vigor, or “heterosis,” in both crop and animal breeding that propelled efforts to dissect its genetic and molecular bases [3]–[10]. Maize geneticists noted early on that inbreeding prior to hybridization drives yield heterosis, and heterotic effects generally improve with greater genetic distance between parental lines [3]. These observations led to the notion that heterosis derives from genome-wide masking of independently accrued deleterious recessive mutations. Extensive quantitative genetic, transcriptomic, and genomic sequencing studies in crop and model plants have provided widespread indirect support for a “dominance complementation” model [2], [6], [11]; however, there is lingering evidence that a model known as overdominance might also contribute to heterosis [5]–[8]. Overdominance has long been an appealing explanation, because theoretically heterozygosity at only a single gene is needed to cause heterotic effects, presumably from intra-locus allelic interactions functionally superseding any one allelic form. However, the relevance of overdominance for yield and whether allelic interactions are the underlying cause remains controversial, primarily because quantitative trait locus (QTL) mapping studies reporting overdominant QTL have failed to pinpoint responsible genes [12]–[16]. Importantly, though, there have been scattered reports of single gene overdominance over the years, and among these have been several unexplained examples from yeast, plants, and animals involving heterozygosity for single gene loss-of-function mutations [17]–[24].

**Figure 1:**
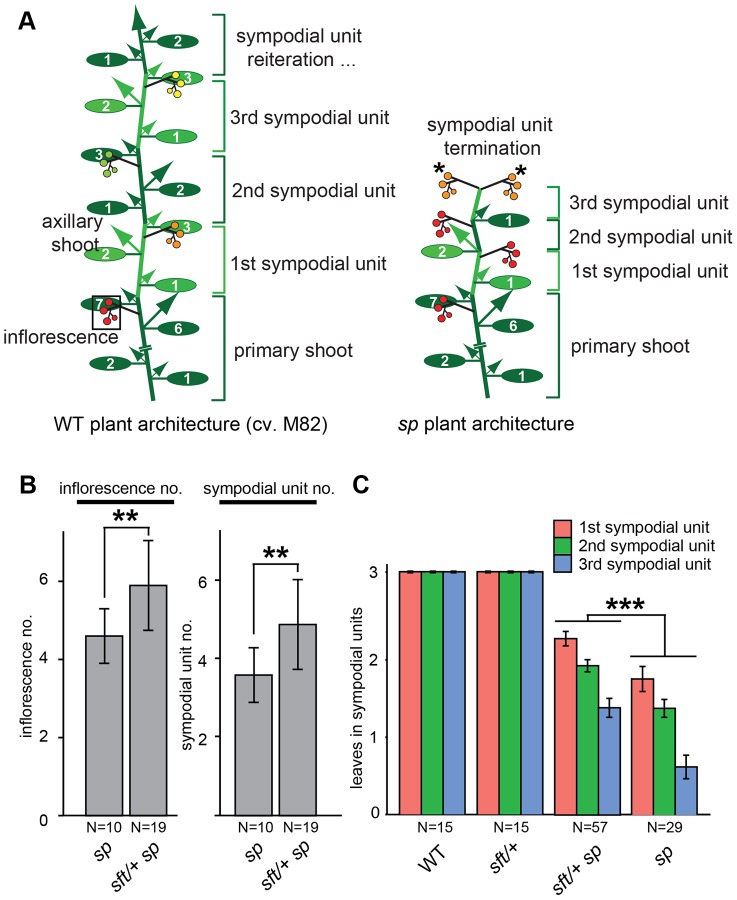
Precocious shoot termination in determinate tomatoes is partially suppressed by *sft/* +** mutant heterozygosity.** (A) Schematic diagrams showing shoot architecture of a wild type (WT) indeterminate tomato plant (left) and an *sp* determinate mutant (right). In WT M82 plants the primary shoot meristem (PSM) from the embryo gives rise to 7–9 leaves before terminating in the first flower of the first multi-flowered inflorescence (boxed). A specialized axillary meristem called a sympodial meristem (SYM) in the axil of the last leaf on primary shoot then generates three leaves before terminating in the first flower of the next inflorescence. In indeterminate tomatoes, this process continues indefinitely (left). In *sp* mutants (right), sympodial cycling accelerates progressively on all shoots causing leaf production to decrease in successive units until growth ends in two juxtaposed inflorescences (asterisks). Alternating colored groups of three ovals represent leaves within successive sympodial units numbered at right. Colored circles represent fruits and flowers within each inflorescence (red: fully ripe fruit; orange: ripening fruit; green: unripe fruit; yellow: flowers) and arrows represent canonical axillary shoots. (B) Compared to *sp* mutants alone, *sft/+ sp* plants produce more inflorescences (left) and sympodial units (right) before sympodial cycling terminates on the main shoot. Genotypes and sample sizes are shown below, and standard deviations of averages are presented. (C) Compared to *sp* alone, *sft/+ sp* plants produce more leaves in the first three sympodial units, indicating a delay in precocious termination. Colored bars indicate average leaf numbers within sympodial units with standard deviations. Statistical significance in B and C was tested by Wilcoxon rank sum test, and significance levels are indicated by asterisks (*P<0.05, **P<0.01, ***P<0.001).

We previously reported a dramatic case of overdominance for tomato yield in multiple environments and planting densities resulting from loss-of-function mutations in the gene *SINGLE FLOWER TRUSS* (*SFT*) encoding the generic flowering hormone florigen [25]. Tomato yield, on both a per plant basis and in the context of tons per acre, depends partly on fruit size, but is mainly driven by the production of dozens of multi-flowered inflorescences and resulting fruit clusters that develop according to the “sympodial” growth habit [26]. The defining feature of sympodial plants is the shoot apical meristem (SAM) ends growth by differentiating into a terminal flower after producing a set number of leaves, and growth then renews from a specialized axillary (i.e. sympodial) meristem (SYM) that, in tomato, produces just three leaves before undergoing its own flowering transition and termination. Indefinite reiteration of three-leaf sympodial flowering events results in an “indeterminate” plant that continuously produces equally spaced inflorescences (Figure 1A). In homozygous *sft* mutants, reduced florigen signals delay the transition to reproductive growth and cause a substantial loss of flower production and yield due to loss of sympodial growth and conversion of inflorescences into leafy vegetative shoots producing scattered flowers [27]. Counter-intuitively, *sft/+* heterozygotes generate more inflorescences, flowers, and harvestable ripe fruits compared to parental controls in the same growing period, but these effects are limited to “determinate” tomato types in which sympodial shoot and inflorescence production ends prematurely due to a classical mutation in the gene *SELF PRUNING* (*SP*) (Figure 1A) [25], [26]. Notably, *SP* is a flowering repressor and a known florigen antagonist in the *SFT* gene family, implying that *SFT-*dependent yield heterosis is likely directly linked to the flowering transition, and specifically to the opposing functional relationship of *SP* to *SFT.*

Tomato breeding goals are multifaceted and shift according to the needs and desires of growers (e.g. improved pest resistances) and consumers (e.g. better quality), but one unwavering aim is to improve yield. Indeterminate cultivars are grown commercially to enable continuous market delivery of “round,” “roma,” “cocktail,” “grape,” and “cherry” tomato types that are eaten fresh and command a premium price. Indeterminate tomatoes are primarily grown in greenhouses where successively ripening clusters are harvested by hand multiple times over an extended period, in some cases up to a year, to maximize yield on plants that must be pruned to one or two main shoots to enable efficient greenhouse growth and maintain fresh market quality [28]. While the necessary pruning of indeterminate tomatoes facilitates agronomic practices that maximize quality, such as size, shape, and flavor, it also limits yield [29]. In contrast, tomatoes grown for sauces, pastes, juices, or other processed can or jar products where fruit quality is less relevant, must be managed agronomically to produce maximum yields (per acre) through once-over mechanical harvests to be economically justified [28]. Maximal yields for processing tomatoes are achieved by growing determinate *sp* mutants in the open field to their full potential, because sequential sympodial shoots transition to flowering progressively faster in *sp* plants, which results in a compact bush-like form where fruits ripen uniformly (Figure 1A) [26]. Thus, *sp* varieties lend themselves to once-over mechanical harvesting and have therefore come to dominate the processing tomato industry, although determinate varieties have also been bred for fresh market production [28]. In a parallel to the physical pruning of indeterminate tomatoes, one drawback of *sp*-imposed determinate growth is that inflorescence and fruit production is restricted, because of a genetic pruning that causes sympodial cycling to stop. Thus, strategies to improve processing tomato yield are limited, primarily because the most logical approach of simply increasing sympodial flowering events would lead back to indeterminate growth and large plants that perform poorly in the field from competition and a loss of uniform ripening. Thus, maximizing inflorescence and fruit production while simultaneously minimizing shoot production for the processing tomato industry has remained a challenging goal. To explore how interactions between mutations in *SP* and *SFT* affect tomato flowering to create a new optimum for fruit yield, we explored tomato *sft* heterosis from a developmental and molecular context of the reproductive transition and its impact on plant architecture and inflorescence production.

## Results

### *sft/+* heterozygosity suppresses sympodial shoot termination in determinate tomatoes

The discovery that *sft/+* heterozygosity in an *sp* background (*sft/+ sp*) dramatically increases fruit production while only modestly increasing plant size was remarkable, but explaining this single gene overdominant effect was limited to showing that the yield boost mostly came from *sft/+ sp* plants having altered sympodial architectures that lead to more inflorescences [25]. *sft* mutant phenotypes are epistatic over *sp* [27], leading us to speculate that having only one functional allele of *SFT* might result in a dose-dependent partial suppression of *sp* determinacy. Indeed, heterosis disappears in a functional *SP* background [25]; yet, how the *sft/+ sp* genetic constitution affects the flowering process to create a new optimum for yield has not been resolved. To address this, we grew *sp* and *sft/+ sp* plants in controlled greenhouse conditions to precisely compare inflorescence production and flowering times of recurring sympodial shoots on the main axis (i.e. derived from the primary shoot; Figure 1A). We found an average of 1.5 more inflorescences and sympodial units on *sft/+ sp* plants, confirming a delay in sympodial termination (Figure 1B). To determine whether this was based on a delay in the flowering transition of each sympodial shoot, we measured leaf number in the first three units and observed a modest, but significant, increase in leaf production (Figure 1C). Importantly, and as expected [25], these delays required the *sp* background, as *sft/+* heterozygosity alone produced three-leaf sympodial units like WT (Figure 1C). Importantly, delays in flowering time and sympodial termination were also observed on side shoots (Figure S1A–C), indicating a whole plant effect from *sft/+* heterozygosity that explains the increase in total inflorescence number (Figure S1D) [25]. Thus, postponement of sympodial termination in *sp* mutants from *sft/+* heterozygosity is based on recurring weak delays of all main and side shoot sympodial flowering transitions.

### *sft/+* heterozygosity weakly delays the primary flowering transition

**Figure 2:**
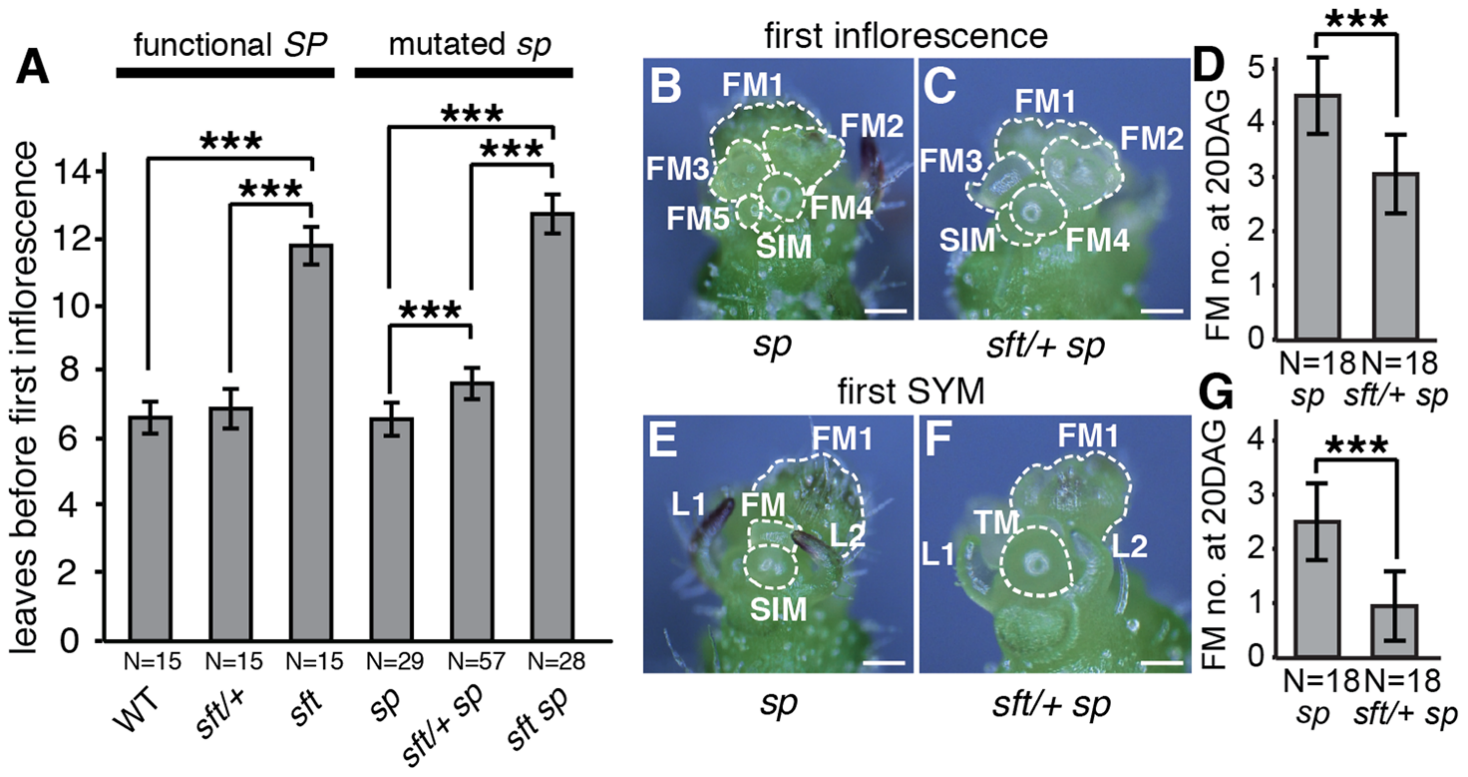
*sft/*+ heterozygosity induces weak semi-dominant delays in both primary and sympodial flowering transitions. (A) *sft/+ sp* plants show slightly delayed primary shoot flowering time compared to *sp* as measured by leaf production before formation of the first inflorescence. Note the extremely delayed flowering of *sft sp* double mutants, indicating a weak semi-dominant effect for *sft/+* heterozygosity. Bars indicate average leaf numbers with standard deviations. Genotypes and sample sizes are shown below. Statistical differences were tested by Wilcoxon rank sum tests and significance levels are marked by asterisks (***P<0.001). (B–G) Representative images and quantification of developmental progression (ontogeny) of meristems in the first inflorescence and sympodial shoot meristems (SYM) of *sp* (left images) and *sft/+ sp* plants (right images) at 20^th^ DAG. Both *sp* (B) and *sft/+ sp* (C) PSMs have completed the primary flowering transition and generated a series of floral meristems (FM) and sympodial inflorescence meristems (SIM) [26], [30]. *sft/+ sp* plants are consistently one SIM behind ontogenically, consistent with a weak delay in flowering from *sft/+* heterozygosity (D). Developmental progression of the first SYM in *sp* (E) and *sft/ + sp* (F) plants at the same time point as in B–C. While the SYM of *sp* mutants has already completed the flowering transition and differentiated into the first or second FM and initiated the next SIM, the SYM of *sft/+ sp* plants is still transitioning or initiating the first SIM, indicating a developmental delay parallel to the PSM of *sft/+ sp* plants (G). In D and G, bars indicate average numbers of initiated FMs with standard deviations. Genotypes and sample sizes are shown below. Statistical differences were tested by Wilcoxon rank sum tests and significance levels are marked by asterisks (***P<0.001). Scale bar: 100 um.

Initiation and perpetuation of tomato sympodial growth depends on a gradual flowering transition culminating in PSM termination in a process mediated in part by accumulating florigen product from *SFT* counterbalancing repressive signals from *SP*. Regardless of whether *SP* is mutated, mutations in *SFT* cause late flowering and produce vegetative inflorescences, and strong alleles fail to initiate sympodial growth (Figure 2A) [27]. Our observation that precocious sympodial termination was delayed in *sft/+ sp* plants beginning with the first sympodial shoot (Figure 1C) led us to ask whether the flowering delay might commence in the PSM where *sft* homozygous mutant phenotypes first manifest. Surprisingly, whereas flowering time of *sft/+* heterozygotes alone was not significantly different from *sp* mutants and WT, *sft/+ sp* plants were slightly later flowering (Figure 2A). We pinpointed this weak semi-dominant effect more precisely by evaluating developmental progression (ontogeny) of meristems. Like vegetative shoots, multi-flowered inflorescences of tomato are based on sympodial growth [26]. Just before the PSM transitions to a terminal floral meristem (FM), a sympodial inflorescence meristem (SIM) initiates perpendicularly, and this process reiterates several times to produce the characteristic zigzag inflorescence [30]. At 20 days after germination (DAG), we quantified SIM production in the primary inflorescence and found that *sft/+ sp* plants were on average one SIM behind *sp* mutants (Figure 2B–D). At this same point, while the first SYM of *sp* plants had already given rise to the first or second FM-SIM pair of the second inflorescence, most *sft/+ sp* SYMs were still in the reproductive transition (no FM evident morphologically) or starting the development of the first SIM-FM pair (Figure 2E–G). Thus, having only one fully functional allele of *SFT* delays the flowering transitions of both primary and sympodial shoots in *sp* mutants.

### *sft/+* heterozygosity delays seedling development and primary shoot meristem maturation

**Figure 3:**
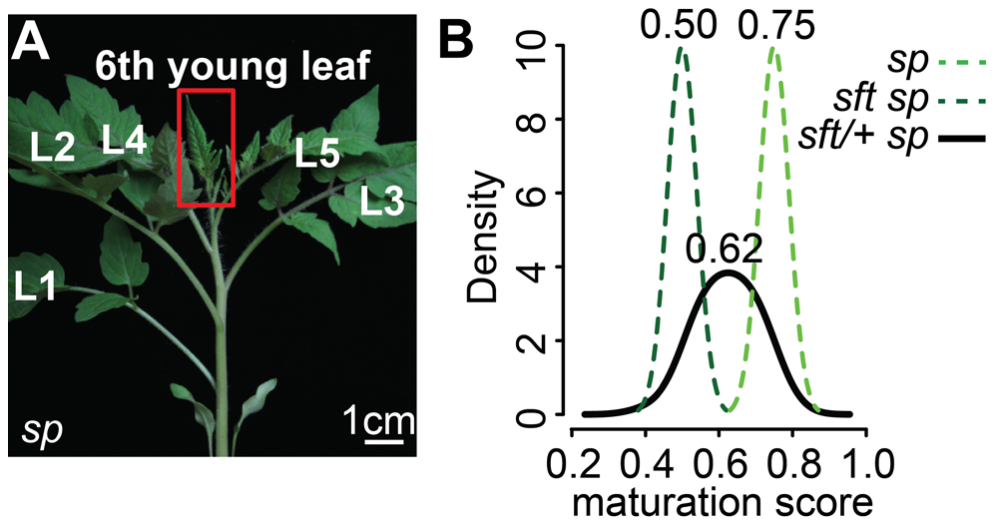
Transcriptome profiling reveals an early semi-dominant delay on seedling development from *sft/*+ heterozygosity. (A) Representative 6^th^ expanding leaf from *sp* mutants. The same leaf and stage (3 cm long) was profiled by RNA-Seq for *sft/+ sp* and *sft sp* genotypes. (B) Molecular quantification of leaf maturation using the DDI algorithm [31]. Given that seedling development of *sft sp* is delayed compared to *sp* based on extreme late flowering, the *sft sp* 6^th^ expanding leaf was designated an early leaf calibration point. Dark and light green curves indicate *sft sp* and *sp* maturation score distributions based on 124 DDI-defined marker genes. The black curve for the *sft/+ sp* 6^th^ leaf indicates an intermediate maturation state. Numbers above indicate average maturation scores.

Our developmental findings suggested that *sft/+* overdominance and yield increases might commence with a semi-dominant delay of the primary flowering event. The flowering transition is paralleled by a maturation of seedlings marked by changes in morphological complexity and molecular states (e.g. transcriptomes) of leaves [27], [31]. As leaves of *sft/+ sp* plants are indistinguishable from those of WT and *sp*, we captured global gene expression patterns of the 6^th^ expanding (3 cm) leaf, which is when differences in meristem ontogeny first appear (Figure 2B–G, Figure 3A and Dataset S1). In comparing *sp* single and *sft sp* double mutant leaf mRNA-Seq generated transcriptomes with those of *sft/+ sp* plants, we found 838 differentially expressed genes among all genotypes. Previous studies comparing gene expression between hybrids and parents involved whole genome heterozygosity and reported thousands of differentially expressed genes representing all modes of gene action (e.g. dominant, recessive, additive, overdominant, etc.) [6], [8], [32]. Surprisingly, despite having heterozygosity at only a single gene in an otherwise homozygous background, we observed expression changes in all directions (Dataset S2). One possible explanation among many for this complexity is that *SFT* is involved in multiple feedback loops and regulates major signaling cascades [33]. However, our primary interest was not to classify and compare these expression differences to whole genome heterozygotes or to dissect transcriptional regulatory networks controlled by *SP* or *SFT*, but rather to use the RNA-Seq data as a quantitative molecular phenotyping tool to determine if there are changes in seedling maturation caused by *sft/+* heterozygosity before gross morphological differences in shoot architecture become apparent.

**Figure 4:**
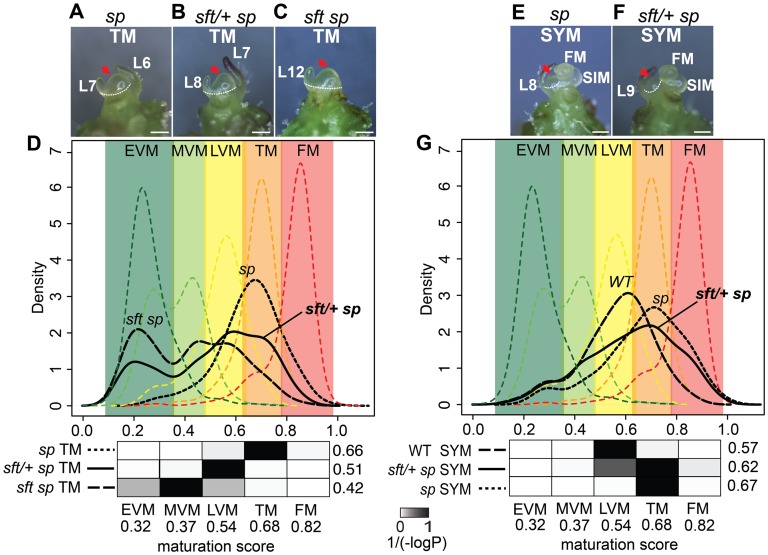
Transcriptome profiling reveals a semi-dominant delay in meristem maturation from *sft/*+ heterozygosity. (A–C) Stereoscope images showing morphology and dissection (white dashed line) of the TM stage used for mRNA-Seq from *sp* (A), *sft/+ sp* (B) and *sft sp* (C) genotypes. Scale bar: 100 um. Red arrows highlight identical TM morphologies. L: leaf primordium number. The additional leaf primordium at the *sft/+ sp* TM is consistent with the one leaf delay in primary shoot flowering time (Figure 2). (D) DDI quantification of maturation scores for *sp*, *sft sp*, and *sft/+ sp* predicted from the WT PSM meristem maturation atlas [34]. Colored dashed curves indicate maturation stages for the 5 PSM stages used for calibration EVM, MVM, LVM, TM and FM [the Early, Middle, and Late Vegetative Meristems, Transition Meristem and the Flower Meristem]. Colored areas define boundaries of these stages estimated from the curves. Maturation scores are derived from 637 DDI-selected marker genes (Dataset S3). Student's t-tests are presented as heat-maps of scaled 1/(−log_10_ *P*) values below each graph, and associated numbers to the right indicate average maturation scores for the predicted meristems. Darker color indicates greater similarity in maturation state. Note the statistically intermediate TM maturation state of *sft/+ sp* relative to *sft sp* and *sp*, indicating *sft/+* heterozygosity causes a semi-dominant delay in the primary flowering transition. The presence of more than one peak along the curves of the *sft sp* and *sft/+ sp* genotypes reflect mixed maturation states for these TMs, as different subsets of marker genes are driving different maturation stage estimates that translate to less uniform maturation patterns. (E–F) Stereoscope images showing morphology and dissection of the first sympodial shoot meristem (SYM) used for mRNA-Seq profiling in *sp* (E) and *sft/+ sp* genotypes (F). Meristems and leaf primordia are marked as in Figure 2. (G) DDI quantification of SYM maturation scores from *sp*, *sft/+ sp*, and WT using the PSM stages as calibrations. Maturation scores for *sft/+ sp*, *sp* and WT indicate an intermediate maturation state for the SYM of *sft/+ sp* plants, mirroring the delay in the PSM. P-value heat maps are shown below along with average maturation scores to the right.

The Digital Differentiation Index (DDI) algorithm identifies transcriptional marker genes whose expressions peak at chosen reference stages to identify stage-enriched marker genes and then queries these marker genes from transcriptomes of “unknown” tissues to predict their maturation states relative to the references [31]. DDI revealed that *sft/+ sp* 6^th^ leaf maturity was in between *sft sp* and *sp*, indicating that *sft/+* heterozygosity delays maturation of *sp* plants already as young seedlings (Figure 3B, Dataset S3). We next asked whether the change in *SFT* dosage might be sensed in the PSM before it transitioned to flowering. We previously captured and quantified transcriptomes of five developmental stages of PSM maturation, which revealed a meristem maturation clock underlies a gradual transition of the PSM to a reproductive state [34]. The transition meristem (TM) stage of this clock is marked by increasing expression of flowering transition genes [34], and we therefore chose this stage for molecular phenotyping and comparison (Dataset S2 and S3). Importantly, TMs can be collected at precisely matched ontogenetic points, defined by initiation of the last leaf and indistinguishable meristem morphologies of tall round domes (Figure 4A–C) [34]. As expected based on the primary inflorescence of *sft* mutants reverting into a vegetative shoot, and consistent with *sft* epistatic over *sp*, DDI revealed that the TM of *sft sp* double mutants exhibited a severely delayed maturation, most closely matching a vegetative meristem state (Figure 4D). In contrast, whereas *sp* TM maturity was indistinguishable from WT, the *sft/+ sp* TM was delayed relative to *sp* and therefore intermediate between *sp* single and *sft sp* double mutants (Figure 4D). Importantly, we also profiled the first SYM from *sp* and *sft/+ sp* plants (*sft sp* plants fail to form a SYM) (Figure 4E and F), and found that, like in the PSM, the *sft/+ sp* SYM was also delayed relative to *sp* (Figure 4G). Altogether, these expression data suggest an early semi-dominant effect on the PSM flowering transition is the triggering event for *sft/+* yield increases, and that all subsequently formed vegetative meristems in *sp* plants become equally sensitive to reduced dosage of *SFT* as they transition to a reproductive state.

### Suppression of *SFT* by artificial microRNA phenocopies the dosage effects of *sft/+* heterozygosity

**Figure 5:**
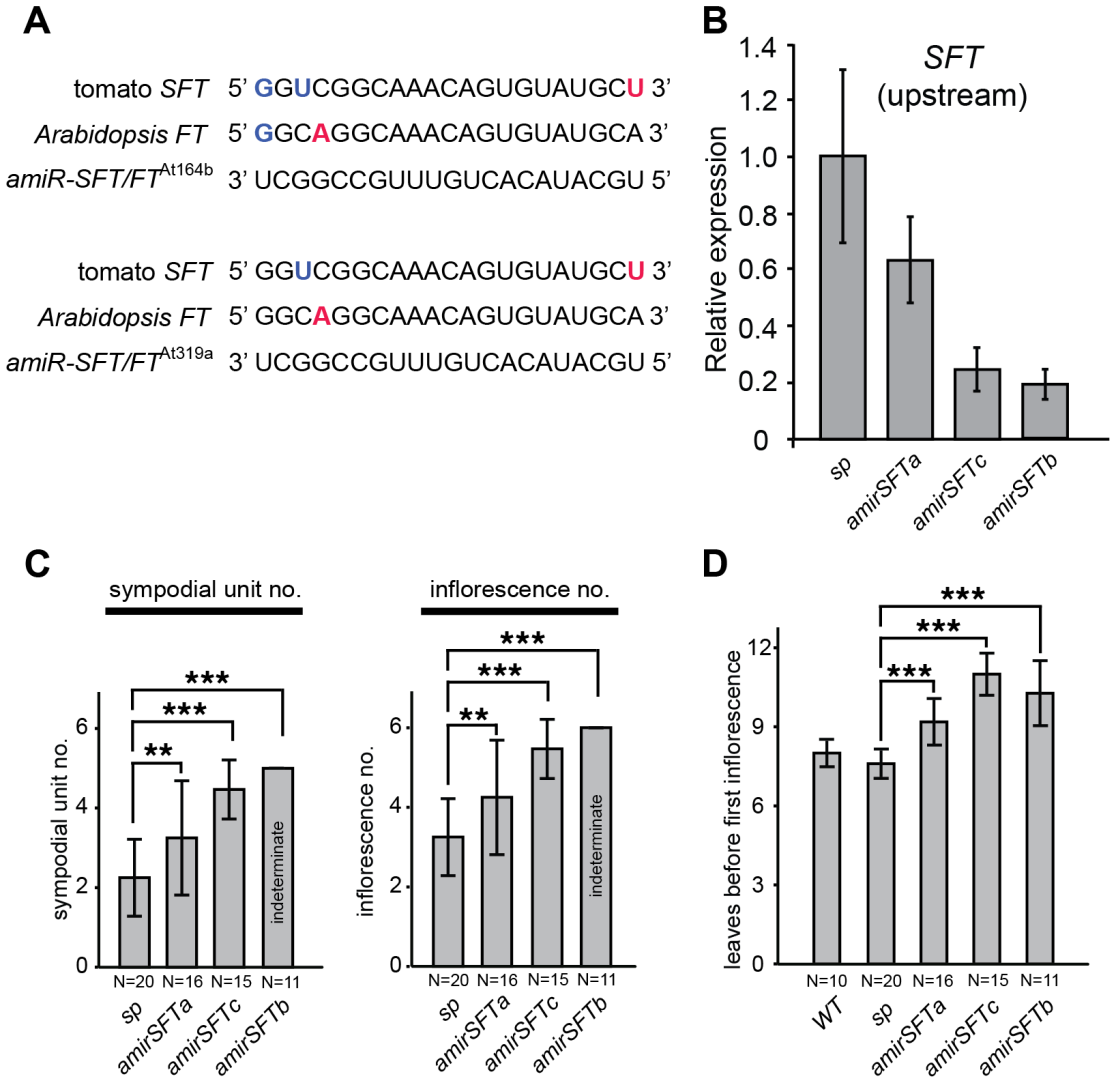
Reducing *SFT* transcripts with artificial microRNAs mimics the dosage effects of *sft/*+ heterozygosity. (A) Artificial microRNAs targeting tomato *SFT* and *Arabidopsis FT*. Shown are alignments of amiR-SFT/FT*^At^* ^164b^ and amiR-SFT/FT*^At^* ^319a^ with the complementary region of *SFT* and *FT*. G–U wobbles and mismatches between the two amiR-SFT/FTs and the target are highlighted in the target sequence with bold blue and red, respectively. (B) Quantitative RT-PCR measurements of tomato *SFT* transcript levels in *amirSFT* plants showing knock down. Results shown are from using primers targeting *SFT* transcripts 5′ to the amiRNA binding site, consistent with reports of primer-dependent transitivity occurring at the 3′ to 5′ direction upon the initial target cleavage, resulting in degradation of the 5′ cleaved product of the target but not the 3′ product [80], [81] (Figure S2). Bars indicate relative expression level and error bars indicate standard deviation among replicates. (C) Depending on the strength of suppression, *amirSFT* plants produce at least one additional sympodial unit and inflorescence compared to *sp* alone, indicating that reducing *SFT* transcript levels by artificial microRNA partially suppresses *sp* sympodial termination, mimicking the dosage effect of *sft/+* heterozygosity. Note that some *amirSFTc* progeny plants showed indeterminacy, whereas *amirSFTb* progeny plants were always indeterminate, indicating that a stronger suppression of *SFT* completely suppresses the *sp* phenotype and reverts the plants to normal sympodial cycling. Differences in sympodial unit and inflorescence numbers between *amirSFT* and *sp* plants were tested by Wilcoxon rank sum test and significance levels are marked by asterisks (* P<0.05, ** P<0.01, *** P<0.001). (D) *amirSFT* plants have delayed primary shoot flowering time compared to *sp* and WT controls, similar to *sft/+* heterozygosity. Bars indicate average leaf numbers with standard deviations. Genotypes and sample sizes are shown below. Differences in leaf numbers between *amirSFT* and *sp* plants were tested by Wilcoxon rank sum test and significance levels are marked by asterisks (* P<0.05, ** P<0.01, *** P<0.001).

Our findings that *sft* single gene overdominance traced back to cumulative delays on recurring flowering transitions led us to reason that the dosage effects of *sft/+* heterozygosity might be recapitulated by simply partially reducing levels of functional *SFT* transcripts. We tested this by over-expressing artificial microRNAs against *SFT* (*35S::amirSFT*) in the *sp* background [35], [36]. In addition to *SFT*, the artificial microRNAs were designed to target the *Arabidopsis thaliana SFT* ortholog, *FLOWERING LOCUS T* (*FT*), to assess their broad efficacy, and were incorporated into two different *Arabidopsis* pre-microRNA templates, *At* pre-mir164b and *At* pre-mir319a, to guard against differential amir backbone efficiencies (Figure 5A). In *Arabidopsis*, *35S:amiR-SFT/FT^At164b^* and *35S:amiR-SFT/FT^At319a^* transformants exhibited late flowering phenotypes equivalent to *ft* mutants (Figure S2B–C). In tomato, six of eight first generation (T1) transformants showed *sp* suppression phenotypes, and we selected three lines representing the range of observed suppression for further analysis. *SFT* transcript abundance was evaluated in these lines by quantitative RT-PCR, revealing a range of knockdown levels by the artificial microRNAs (Figure 5B). We evaluated progenies from two *35S:amiR-SFT/FT^At164b^* (referred to as *amirSFTa* and *amirSFTb*) and one *35S:amiR-SFT/FT ^At319a^* (referred to as *amirSFTc*) transformants, and found that the *amirSFTa* produced an average of one additional sympodial unit and inflorescence compared to non-transformed *sp* mutants, closely resembling the dosage effects of *sft/+* heterozygosity (Figure 5C). *amirSFTc* showed greater suppression, terminating sympodial growth after producing often more than two additional units, while *amirSFTb* fully suppressed *sp* to indeterminacy like WT plants (Figure 5C). Notably, the level of suppression of *sp* determinacy corresponded with the level of knockdown of *SFT*; e.g. the indeterminate line, *amirSFTb*, showed the greatest reduction of *SFT* transcripts (Figure 5B–C). In all six lines, we failed to find strong *sft sp* double mutant phenotypes of reverted inflorescences or loss of sympodial growth, suggesting only weak alleles of *SFT* were created with the *35S::amirSFT* transgene – an effect that is also consistent with often observed weak target knockdown by artificial microRNAs [35], [36]. Importantly, we found delayed flowering time in successive sympodial units like in *sft/+ sp* heterozygotes, and all three *amirSFT* progeny populations exhibited delayed primary shoot flowering time (Figure 5D). Thus, tuning *SFT* dosage transgenically mimics the effects of *sft/+* heterozygosity, further illustrating that a classical epistasis relationship between the *sft* and *sp* mutants is ultimately responsible for the overdominant effect on yield.

### A dosage effect from florigen mutant heterozygosity is conserved in *Arabidopsis*, but does not cause heterosis

**Figure 6:**
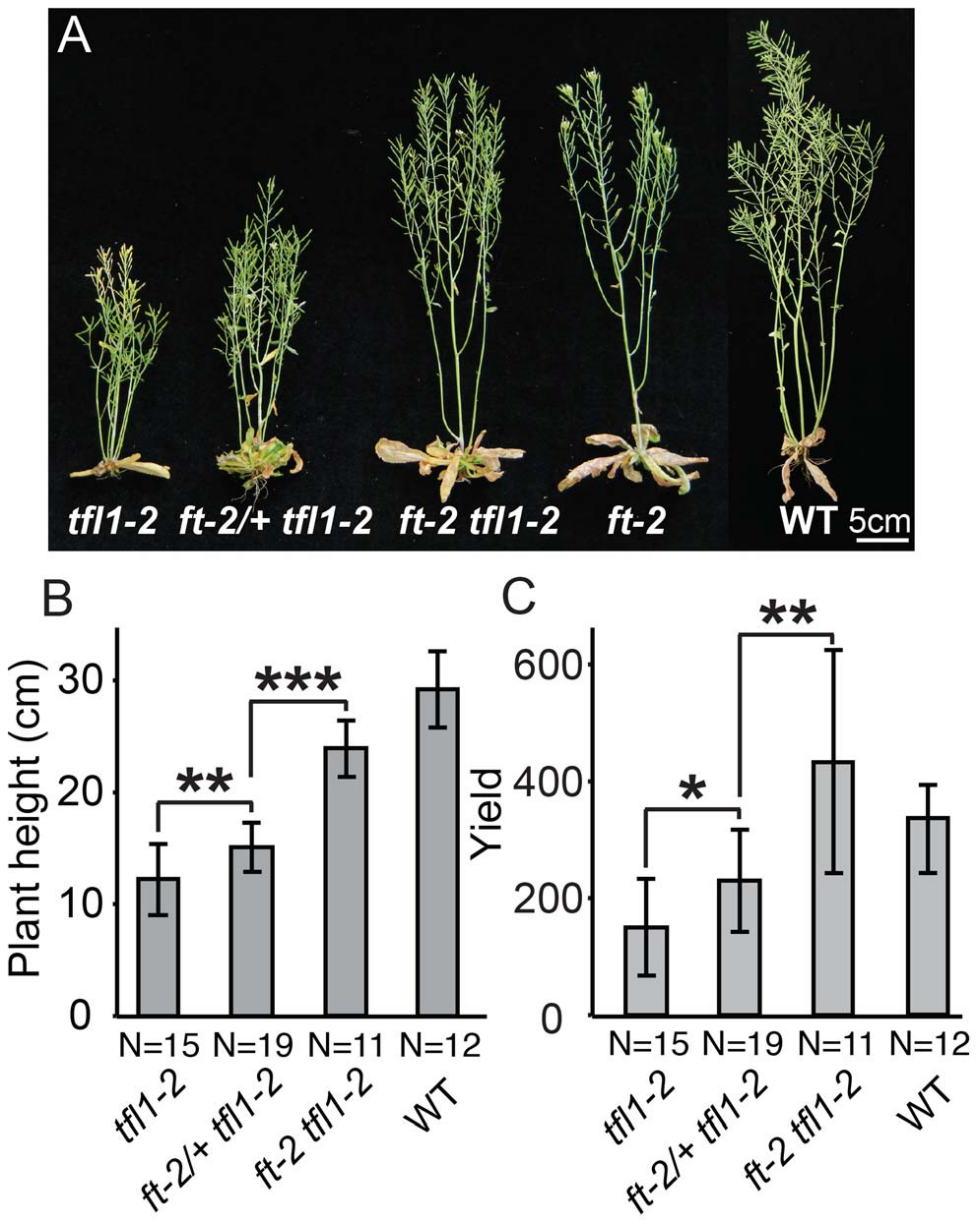
Dose-dependent suppression of *tfl1* (*sp*) by *ft/*+ (*sft/*+) heterozygosity is conserved in *Arabidopsis thaliana*. (A) Representative plants from left to right of: *tfl1-2* single mutants, *ft-2/+ tfl1-2*, *ft-2 tfl1-2* double mutants, *ft-2* single mutants and wild type *Ler-0* (WT) showing the intermediate height of *ft-2/+ tfl1-2* plants compared to *tfl1-2* and *ft-2 tfl1-2* genotypes. (B–C) Statistical comparisons among all genotypes for plant height and flower/fruit yield showing semi-dominant effects from *ft-2/+*heterozygosity in the *tfl1-2* background. Bars indicate average values with standard deviation. Genotypes and sample size are shown below. Differences between genotypes were tested by a Wilcoxon rank sum test and significance levels are marked by asterisks (*P<0.05, **P<0.01, ***P<0.001). (B) *ft-2* heterozygosity in a *tfl1-2* mutant background partially suppresses the early flowering and early termination phenotype of the *tfl1-2* mutation in a semi-dominant manner, resulting in plant height in between *tfl1-2* and *ft-2 tfl1-2* mutant parental lines. (C) Unlike tomato, *ft/+* heterozygosity in a *tfl1-2* mutant background does not drive heterosis for yield (number of total siliques and floral buds) in *Arabidopsis*. Rather, yield in the *ft-2/+ tfl1-2* plants is intermediate to *tfl1-2* and *ft-2 tfl1-2* double mutants.

As florigen is a universal inductive signal for flowering that several flowering pathways converge upon [37], [38], we wondered if and how florigen mutant heterozygosity in a different system might affect growth, and specifically whether heterosis would result. We tested this by creating orthologous mutant combinations in *Arabidopsis thaliana*, which is a monopodial plant in which a single flowering event converts the SAM into a continuously growing inflorescence meristem (IM) that produces flowers laterally, in contrast to the tomato sympodial growth habit in which multiple flowering transitions occur. Despite this difference, *Arabidopsis ft* (*sft*) mutants are likewise late flowering [39] and completely epistatic over the early flowering and precocious termination of inflorescence meristems of *tfl* (*sp*) mutants [40]. To evaluate potential dosage effects of *ft/+* heterozygosity, we phenotyped progeny from *ft-2/+ tfl1-2* plants, in which the *ft-2* mutation, a strong allele, segregates in the *tfl1* background (Figure 6A). We measured flowering time by counting rosette leaves and found a clear dosage effect in *ft-2/+ tfl1* plants compared to *tfl1* single and *ft-2 tfl1* double mutants (Figure S3A). We next tested for heterosis by quantifying yield related traits, including plant height, number of axillary shoots, and, as a parallel to tomato yield, the number of siliques, flowers, and flower buds (Figure 6B–C and Figure S3B–D). Surprisingly, *ft/+ tfl* plants showed semi-dominance for plant height and total yield (Figure 6B and C), and similar effects were observed for a moderate second allele of *ft* (Figure S3E). Thus, whereas the dosage effect on flowering time from florigen mutant heterozygosity is conserved in the monopodial growth habit of *Arabidopsis*, it does not translate to heterosis.

## Discussion

Crop yields derive from a complex integration of fitness-related traits founded on developmental and physiological mechanisms for organ production and biomass accumulation. Thus, studying heterosis inevitably involves a broad analysis of the myriad mechanisms controlling plant growth. It is therefore perhaps not surprising that recent gathering of vast genetic, phenotypic, and molecular data on cases of heterosis from diverse systems has suggested that multiple non-mutually exclusive system-specific mechanisms are likely at work [8]–[10], [41]. Looking at heterosis from the developmental perspective, it would be reasonable to assume *a priori* that flowering would have a major role given that selection of allelic variation for flowering time regulators has been a major contributor to adaptation, domestication, and maximizing crop yields through classical and modern breeding [42]. In rice, for example, alleles of strong effect from various flowering regulators, many showing epistatic interactions, were selected to enable growth at different climates and day lengths [43]–[45]. The same was achieved in maize, but, instead, dozens of loci of small additive effect were found to be involved [46]. In both rice and maize, and as occurred during the domestication and breeding of many crops, this selection enabled a shift from an extended period of flowering in wild populations to uniform flowering, which provided sudden bursts of yield that facilitated agronomic practices, particularly harvesting [42]. Interestingly, the genetic path leading to high yielding tomatoes has differed from other major crops in that domestication has mostly acted on fruit size to increase yield with little evidence for selection on flowering [47]–[50]. Indeed, while there is certainly flowering time and architectural variation among distantly related wild tomato species [51], cultivated tomatoes and their wild progenitor, *S. pimpinellifolium*, share nearly identical flowering times and indeterminate growth habits, suggesting there was little or no standing genetic variation for artificial selection to act upon [52]. Only with the relatively recent discovery of *sp* did a change in flowering provide a major agronomic shift in how tomato was grown in the field, enabling a burst of flower production and yield on compact plants grown at high density, which gave rise to the processing tomato industry [26]. In this regard, in contrast to maize where altered flowering times are frequently observed in hybrids [10], [53], cultivated tomato hybrids do not differ substantially from their parental inbreds for flowering time, inflorescence production, or overall plant architectures. Only upon introgressing quantitative trait loci (QTL) from distantly related wild species are heterotic effects on yield observed, a subset of which have been tied to changes in flowering and plant architecture, but the causative genes have not been identified [54]. Thus, our dissection of *sft* heterosis is the first to expose a direct link to flowering and resolve the underlying mechanism.

Our combined developmental and molecular phenotyping of *sft/+* overdominance has exposed a novel principle for how tomato plant architecture and yields might be further optimized by taking advantage of the surprising and remarkable level of dosage sensitivity within florigen and the florigen pathway. The genetically induced reduction in dosage of florigen from *sft/+* heterozygosity causes a slight delay in the transition to reproductive growth that, in the context of recurring flowering events of the sympodial habit and the *sp* mutant background, translates to cumulative overdominance. Indeed, this heterosis example, like many others [20]–[24], [55], is conditional. Yet, it is this genetic and developmental conditionality that suggests *sft/+* heterosis could be considered less about heterozygosity and heterosis *per se* and more about the potential to genetically fine-tune *SFT* expression levels to manipulate yield in a way that domestication and breeding efforts have not yet capitalized on, perhaps because standing allelic diversity for florigen and members of its pathway is limited. In this respect, we propose that additional directed quantitative manipulation of the relative doses of *SFT* to *SP* might enable further fine-tuning of flowering, sympodial cycling, and inflorescence production. For example, as yet undiscovered, or artificially created [56], [57], transcriptional or loss-of-function alleles of *SFT* and *SP* of various strengths could be combined in different genetic constitutions to pinpoint an even higher optimum of plant architecture to maximize yield. In an even simpler scenario, homozygosity for very weak mutant alleles of *SFT* in a strong *sp* background, or homozygosity for weaker mutant alleles of *SP* alone, could potentially match or exceed fruit production of the *sft/+ sp* genotype. Finally, beyond tweaking *SP* and *SFT*, partial suppression of *sp* determinacy by generating mutations in other pathway genes, especially those encoding components of the florigen activating complex [58], could provide novel alleles and breeding germplasm that natural variation might not be able to provide.

Importantly, although there is tremendous diversity among angiosperms in when and where inflorescences and flowers form, the *SFT/SP* system is highly conserved [38], [59], [60], suggesting the aforementioned concepts could be applicable to other plants. Yet, our findings in *Arabidopsis* imply that while dosage effects on flowering time from florigen mutant heterozygosity will be broadly conserved, yield benefits might not be, and species-specific outcomes will likely trace back to differences in growth habits. The lack of meristem termination and recurring flowering events in the monopodial growth habit of *Arabidopsis* means that florigen mutant heterozygosity is sensed only once during development, and that no compounding of the semi-dominant dosage effect is possible. Indeed, increasing yield in *Arabidopsis* simply requires a larger plant, which can be achieved by delaying and prolonging flowering either environmentally through short day growth conditions or genetically through mutations in flowering regulators like *FT*. Consistent with this, we found that homozygous *ft* mutants were the highest yielding of all genotypes (Figure 6). At first glance, this would suggest limited possibilities for exploiting our findings beyond tomato; however, for some breeding goals, such as improving biomass, delaying flowering quantitatively and predictably through an allelic series of florigen mutants in either the homozygous or heterozygous condition could prove valuable to customize plant architecture and size for particular agronomic needs. Remarkably, yield benefits from heterozygous mutations in florigen orthologs have been found in at least one plant that lacks sympodial growth. In a strikingly similar example to tomato, a major domestication QTL for flowering time in sunflower traces back to a deletion in a duplicated paralogous *FT* gene that causes heterosis for both seed size and weight when heterozygous under short day conditions [61]. In another example, a classical report of overdominance for sorghum yield involves heterozygosity for an as yet uncharacterized late flowering mutant that has all the hallmarks of being defective in florigen or a florigen pathway component [62]. Thus, heterozygosity for florigen mutants holds potential for broadly improving crop yields, which, in hindsight, is perhaps not surprising given that selection for beneficial alleles of various strengths in florigen family genes, especially orthologs of *SFT* and *SP*, was key for the domestication of barley [63], beets [64], beans [65], [66], grape [67], potatoes [68], roses [69], soybeans [70], [71], sunflower [61], tobacco [72], and likely many other plants. With these examples in mind, and considering our findings in *Arabidopsis*, we suggest that *sft/+* heterozygosity in a dose-dependent epistatic relationship with *sp* may represent only one of several ways to genetically tailor florigen levels, and that hunting for new alleles in existing germplasm or engineering custom alleles could allow an optimal fine-tuning of florigen and its pathway to maximize flowering, inflorescence production, and other yield components in these and other crops. The potential to broadly manipulate agronomic traits by florigen and its family members in diverse plant species stems not only from roles in flowering time, but also as general coordinators of diverse physiological processes affecting multiple aspects of plant growth and fertility [38]. Thus, parallel to how mutations in biosynthesis genes for the hormone gibberellin created the dwarf mutants that propelled the Green Revolution [73], our findings provide compelling evidence that manipulating florigen family genes can provide a new path to meet current breeding challenges associated with a rapidly changing climate.

## Materials and Methods

### Tomato plant growth conditions, genotyping, and phenotyping

The *sp* mutant was first reported more than 80 years ago and arose spontaneously, and the strong *sft* mutant allele used in this study, *sft-7187*, was isolated from a fast neutron mutagenesis screen performed in tomato cultivar M82, and has a two nucleotide deletion that truncates the C-terminal portion of the protein [26], [27], [74]. All mutants were backcrossed to M82 at least four times to eliminate background mutations prior to the original yield trials [25]. For all experiments in this study, plants were grown in controlled greenhouse conditions at Cold Spring Harbor Laboratory. Greenhouses were supplemented with artificial light from high-pressure sodium bulbs (50 µmol/m2/sec; 16 h/8 h) and daytime temperature was 78°F and nighttime temperature was 65°F, with a relative humidity of 40–60%. Tomato F2 generation seeds derived from self fertilization of an *sft/+ sp* F1 plant were grown in 72-cell insert flats and transplanted after four weeks into 2 gallon pots (three plants per pot) for quantitative phenotyping. Young leaf tissue was collected from each F2 individual at the time of transplanting for DNA extraction and genotyping. Total genomic DNA was extracted using a standard cetrimonium bromide (CTAB) DNA extraction protocol. Genomic fragments of the *SFT* locus were amplified using the PCR primers: “sft-7187 full exon F2” 5′-GGGCAAGAAATAGTGAGCTAT-3′ and “sft-7187 full exon R2” 5′-TTCAAATAAATTGAGAGGAAGA-3′ and the following PCR program: initial denaturation at 94°C for 3 minutes, then 35 cycles at 94°C for 30 seconds, annealing at 52°C for 30 seconds, extension at 72°C for 1 minute, and a final extension at 72°C for 10 minutes. The PCR products were subjected to enzyme digestion with *Tse*I at 60°C for 6 hours, resulting in two bands for wild type, one band for *sft* mutant and three bands for *sft/+* after running on a 3% agarose gel at 150 V for 40 minutes. The number of leaves in the primary shoot prior to the first inflorescence and leaves within three successive sympodial units were counted for each individual at 8–12 weeks after germination. This same phenotyping scheme was applied to two axillary shoots: the lower (basal) axillary shoot originating from the axil of the first leaf on the primary shoot and the uppermost (proximal) axillary shoot originating from the axil of the last leaf formed before the first inflorescence. Quantitative measurements for inflorescence number, sympodial unit number, primary and lateral shoot flowering time, and leaf number in three sympodial units were evaluated for the shape of each phenotype's distribution and subjected to two-tailed Wilcoxon rank sum tests between genotypes and Kruskal–Wallis one-way analysis of variance across all genotypes. To quantitatively compare the progression of sympodial inflorescence meristem (SIM) and floral meristem (FM) initiation on the first developing inflorescence of *sp* and *sft/+ sp* plants, we germinated 18 plants for both genotypes at the same time and counted the number of differentiated FMs on both primary and sympodial shoots at 20^th^ days after germination (DAG). The FM numbers were subjected to two-tailed Wilcoxon rank sum tests between genotypes. To image live meristems, shoot apices were dissected from seedlings, and older leaf primordia (>150 µm) were removed under a Nikon SMZ1500 stereomicroscope. The meristem images were taken immediately after dissection with an integrated Nikon digital camera, recaptured by Z-series manually, and merged to create focused images.

### *Arabidopsis* plant growth conditions, genotyping, and phenotyping

*Arabidopsis thaliana* plants were grown in the greenhouse under long day (16 h light, 8 hr dark) conditions in 32-cell flats with two plants per cell. Individual seeds were delivered to the corner of each cell to avoid growth competition during germination. The seeds were stratified at 4°C for 4 days before transferring to a long day greenhouse maintained at 21°C. All mutant lines were acquired from the Arabidopsis Biological Resource Center (ABRC) and originated from EMS mutagenesis in the Landsberg *erecta* (L*er*) background. Homozygous *tfl1-2* mutant plants were crossed to a moderate (*ft-1*) and strong (*ft-2*) allele of *ft.* Individual F1 plants from each cross were self-fertilized to generate F2 populations segregating for both *tfl1-2* and *ft* mutants. Plants homozygous for the *tfl1-2* mutation and heterozygous for the *ft-2* mutation were self-fertilized to generate F3 populations fixed for the *tfl1-2* mutation and segregating for the *ft-2* mutation. Tissue was harvested from young rosette leaves and DNA was extracted using a standard CTAB DNA extraction protocol. The *tfl1-2* and *ft-2* mutations were detected using derivative CAPS (dCAPS) assays. A fragment of *TFL1* was amplified by PCR using the primers “tfl1-2 dCAPS-F” 5′- AAACGTCTCACTTCCTTTTCCTC-3′ and “tfl1-2 dCAPs-R2” 5′- AAATGAAAAGAAAGAATAAATAAATTAAA**G**GTAC-3′ and a fragment of *FT* was amplified using “ft-2 dCAPS-F2” 5′- CCCTGCTACAACTGGAACAACCTTTGGTG-3′ and “ft-2 dCAPS-R2” 5′- AAACTCGCGAGTGTTGAAGTTCTGG**G**GC-3′. Both *TFL1* and *FT* fragments were amplified using a touchdown PCR program: initial denaturation at 95°C for 3 minutes, then 10 cycles at 95°C for 20 seconds, 65°C for 30 seconds (decreased by −0.5°C/cycle), 72°C for 30 seconds followed by an additional 30 cycles at 95°C for 20 seconds, 52°C for 30 seconds, 72°C for 30 seconds and ending with a final extension at 72°C for 10 minutes. Underlined nucleotides in the aforementioned sequences introduce a new restriction site in the wild type PCR amplicons. *TFL1* PCR amplicons were digested using *Kpn*I for 3 hours at 37°C, which cuts wild type but not mutant sequences. *FT* PCR amplicons were digested using *Hae*III for 3 hours at 37°C, which cuts wild type but not the *ft-2* mutant sequences. Wild type versus mutant banding patterns was resolved on a 3% half MetaPhor agarose-half regular agarose gel. Phenotyping was completed in the F3 generation, and we compared *tfl1-2 ft-2* double, *tfl1-2 ft-2/+* and *tfl1-2* single mutants. Homozygous single mutants and wild type L*er*-0 were grown at the same time for comparison. Phenotyping and imaging was performed when the plants completed flowering and inflorescence meristems stopped growing (6–8 weeks after germination). The height of each plant was measured along the main shoot of the plant from where the base emerged from the rosette to top of the shoot. The number of rosette leaves, axillary shoots, siliques, open flowers, and floral buds were also recorded as measures of flowering time and yield. For each measured trait, the mean and standard deviation was calculated for each genotype. The means were compared using a Student's t-test (Wilcoxon rank sum test when the phenotypic distribution was not normal).

### Global gene expression profiling (mRNA-Seq) of tomato leaves and meristems

Tomato homozygous *sp* mutants, *sft sp* double mutants and F1 single gene heterozygotes of *sft/+ sp* plants were used for leaf and meristem expression profiling experiments. All *sft/+ sp* plants originated from F1 seeds of direct crosses between the *sp* and *sft sp* parents, and a subset of F1 plants were confirmed by PCR genotyping to ensure 100% *sft/+* heterozygosity. Seeds were germinated in petri plates on water-soaked Whatman paper at 28°C for 72 hours until the root radicles emerged. The germinated seeds were then transplanted to 72-cell insert flats with pre-wet soil and placed in the greenhouse. The plants used for leaf expression profiling were transplanted to two-gallon pots (three plants per pot), and tissue from the 6^th^ young expanding leaf from each plant was collected and immediately frozen in liquid Nitrogen when the leaves reached 3 cm in length. Total RNA was extracted using a Qiagen RNeasy mini total RNA extraction kit according to the manufacturer's protocol. Growth of seedlings for meristem expression profiling was monitored daily under a dissecting microscope using the meristem morphological cues marking previously defined maturation stages [34]. At the transition maturation (TM) stage, the cotyledons and leaves were removed from seedlings and the shoot apices with 3 cm hypocotyl attached were collected and stored in 100% acetone followed by vacuum infiltration for 30 minutes. Meristem tissue was dissected from the fixed stems using a surgical blade following the lines shown in Figure 4A–C and E–F under a dissecting microscope after confirming the morphology that marks the TM stage. Total RNA was extracted from the dissected meristem tissues with an Arcturus PicoPure total RNA extraction kit (Life Technologies). Except for the *sp* SYM, which is difficult to capture in high numbers because of a rapid termination, for all genotypes, tissue was harvested and prepared for mRNA-Seq construction for two biological replicates, and *sp* SYM was subjected to two technical replicates. As reported previously [34], two replicates were sufficient to quantify meristem maturation states using the DDI algorithm, which was our primary goal in the expression analysis.

### RNA-Seq library preparation

For all tissues, poly-A containing mRNA was purified from total RNA using Invitrogen oligo-dT DynaBeads for mRNA-Seq library construction using the ScriptSeq v2 RNA library preparation kit (Epicentre). The maximum amount of mRNA input (50 ng) was used when possible to maximize the library output. The final PCR enrichment step was carried out following the standard protocol with 15 cycles and primers with barcode indices supplied by Epicentre to create barcoded mRNA-Seq libraries. The quantity and size distribution of each individual barcoded mRNA-Seq library was detected with a High Sensitivity DNA Chip on a Bioanalyzer 2100 machine (Agilent). The final concentration of each library was verified by qPCR using a KAPA library quantification kit and based on these results, four to six barcoded libraries were pooled together with equal concentration for one lane of Illumina paired-end (PE) 100 bp sequencing on an Illumina HiSeq sequencing machine (Dataset S1). All reads files were deposited to SGN (ftp://ftp.solgenomics.net/transcript_sequences/by_species/Solanum_lycopersicum/libraries/illumina/LippmanZ/) and the mean RPKM values of meristems are visualized on an eFP browser (http://tomatolab.cshl.edu/efp/cgi-bin/efpWeb.cgi, *SFT* heterosis panel).

### Read mapping and analysis

All mRNA-Seq reads were trimmed to 50 bp to remove the bases with low qualities and mapped using Bowtie [75] to the tomato reference CDS [76] with paired-end relationships maintained. Trimming the reads to 50 bp also made the libraries comparable to our previous mRNA-Seq libraries [34] for combined DDI analyses. The lack of size selection step in the Epicentre ScriptSeq v2 mRNA-Seq library preparation protocol allowed lower initial mRNA input but produced a larger insert size range (150 bp∼1000 bp), which lowered the successful mapping with proper distance between paired-end reads. Mapping to predicted CDS also reduced the mapping rate due to failed mapping of reads coming from 5′ and 3′ UTR regions. However, the higher total read number from Illumina HiSeq compensated for the relatively lower mapping rates, yielding comparable mapped read numbers and sequencing depth to previous mRNA-Seq libraries that allowed for differential expression analysis and molecular phenotyping by DDI [34]. The resulting bam alignments were sorted and indexed by SAMtools [77], and the number of reads mapped to each CDS was counted to calculate the raw counts for all libraries. The raw counts from leaf and TM tissues across three genotypes were normalized using the TMM method. The distribution of gene expression levels were modeled following a negative-binomial distribution and tag-wise dispersion were estimated based on two replicates. Finally, exact tests for differential expression were conducted based on the replicates in pairwise comparisons. All normalization and differential expression tests were conducted using the edgeR package [78], [79]. Although only two replicates were performed, we classified gene expression patterns from comparing *sft/+ sp* heterozygotes and homozygous parents into 12 categories belonging to five major classes: additive, recessive, dominant, overdominant and underdominant (Dataset S2) using a threshold of two-fold change and P-value< = 0.01. Numbers of genes in each category were counted and their proportions in each category relative to all differential expressed genes were calculated for the 6^th^ young leaf and TM, respectively, revealing all categories of gene expression changes were detected (Dataset S2).

### Digital Differentiation Index analyses

Raw counts for the leaf expression profiles (including *sp*, *sft/+sp* and *sft sp* 6^th^ young leaves) were incorporated into a master leaf data set. Raw counts for the meristem expression profiles (including *sp* and *sft/+sp* TM and SYM) were incorporated into a master meristem data set that includes all raw counts from our previous meristems profiling experiments [34]. For both master data sets, all raw counts were then summarized over replicates and normalized against number of mapped reads and CDS lengths to calculate RPKM values for DDI analyses [31]. DDI selects samples with known or pre-determined maturation states in the whole data set as calibration points, and then identifies marker genes that show maximum expression at each calibration point. These genes characterize the calibration points molecularly. DDI checks the marker gene expressions in the samples that are submitted to query (the ‘unknown’ samples) and quantifies the ‘unknown’ samples' maturation states relative to the calibration points. For each marker gene, DDI compares expression levels between ‘unknown’ samples and each calibration point and calculates a ‘maturation score’. Collectively, all marker genes generate a distribution of maturation scores for the ‘unknown’ sample [31]. Importantly, curves showing multiple ‘peaks’ reflect a mixed molecular maturation state for the queried tissue, as different marker genes give different maturation estimates. This is most evident in *sft sp* double mutants that still transition to flowering, but at a much slower rate compared to wild type and with vegetative reversion of the inflorescence, indicative of a mixed vegetative-reproductive state. At the same time, a Student's t-test of average maturation score difference between calibration and unknown samples was conducted for each unknown meristem sample, yielding a P-value for the significance of the maturation state difference. For each prediction, this P-value was obtained for comparisons between the unknown sample and temporarily successive calibration points, in order to generate a ‘gradient’ of meristem similarity (plotted in heat-maps in the form of scaled 1/(−log10P)). For example, to predict the maturation state of *sft/+ sp* SYM using the first replicate of WT EVM, MVM, LVM, TM and FM [the Early, Middle, and Late Vegetative Meristems (EVM: 5^th^ leaf initiated; MVM: 6^th^ leaf initiated; LVM: 7^th^ leaf initiated), the Transition Meristem (TM: 8^th^ leaf initiated), and the Flower Meristem (FM)] as calibration points, P-values were calculated for maturation state comparisons SYM vs. EVM, SYM vs. MVM, SYM vs. LVM, SYM vs. TM and SYM vs. FM, respectively. The P-values were then transformed into 1/(−log10P) and scaled across five values into a zero to one range (scaling was done for each prediction independently). Because smaller P-values indicate larger differences in maturation scores, the scaled 1/(−log10P) values quantify the relative similarity of the *sft/+ sp* SYM to each of the five calibration points. With the master leaf data set, DDI analyses were conducted using *sft sp* and *sp* 6^th^ young leaves as two calibration points to predict maturation stages of *sft/+sp* leaf maturation. With the master meristem data set, DDI analyses were conducted using five WT primary shoot meristem (PSM) stages as calibration points to predict maturation stages of *sp*, *sft/+sp* and *sft sp* meristems. As in [34], one replicate of calibration samples was used for marker gene identification (Dataset S3), a second replicate of calibration samples treated as unknowns was predicted and plotted to set the boundaries of maturation stages (colored curves and boxes in Figure 4D and Figure 4G), and averaged RPKM values of predicting leaves and meristems were used to generate and plot the predicted distribution of maturation scores. All parameters for DDI analyses were as previously described [34]. All DDI analyses were carried out using modified R scripts as described previously [34].

### Artificial microRNA construction and transformation

Artificial microRNAs were designed to repress both tomato *SFT* and *Arabidopsis FT* with two different backbones (Figure S1) [35]. The artificial microRNA amiR-SFT/FT*^At^* ^164b^ and amiR-SFT/FT *^At^* ^319a^ were synthesized by DNA2.0 and Bio S&T, respectively, and transformed into both tomato and *Arabidopsis* plants and phenotyped for repression of *SFT* and *FT*, respectively (Figure 5B, Figure S2). Tomato plants carrying mirSFT transgenes were measured for sympodial unit and inflorescence number, and phenotyping stopped after counting five or more sympodial units with two or more leaves in each unit and classified as indeterminate. The means of phenotypes were compared using a Student's t-test (Wilcoxon rank sum test when phenotype distribution is not a normal distribution).

For quantitative RT-PCR of *SFT* transcript abundance in the amirRNA lines, cotyledon tissue was collected from two-week old seedlings for total RNA extraction with Qiagen RNeasy mini total RNA extraction kit including DNase treatment with RNase-free DNase (Qiagen) according to the manufacturer's instructions. First-strand cDNA was then synthesized using the SuperScript III First-Strand Synthesis System with oligo dT (Invitrogen). *Ubiquitin* mRNA (Solyc01g056940) was used as the reference for normalization in quantifying cDNA. 5′ mRNA (upstream, Figure 5B) and 3′ mRNA (downstream, Figure S2A) of *SFT* transcript (Solyc03g063100) from the amirSFT binding site were quantified with 1 ul of cDNA using Phusion High-fidelity DNA polymerase (NEB), iQTM SYBR Green Supermix (Bio-Rad). A loss of transcripts was detected 5′ to the amiRNA binding site, consistent with reports of primer-dependent transitivity occurring at the 3′ to 5′ direction upon the initial target cleavage, resulting in degradation of the 5′ cleaved product of the target but not the 3′ product [80], [81]. Primers pairs used were: 5′-CGTGGTGGTGCTAAGAAGAG-3′ and 5′- ACGAAGCCTCTGAACCTTTC-3′ for *Ubiquitin* (*UBI*); 5′-GCTTAGGCCTTCCCAAGTTA-3′ and 5′-GGGTCCACCATAACCAAAGT-3′ for *5′ mSFT* (upstream); 5′-GACAATTAGGTCGGCAAACA-3′ and 5′-AGCAGCAACAGGTAAACCAA- 3′ for *3′mSFT* (downstream). Two biological replicates of qRT-PCR were performed on the CFX96TM Real-time PCR System (Bio-Rad). qRT-PCR data were calculated from the number of PCR cycles needed to reach the linear phase for each *SFT* transcript from amirSFT lines and normalized against *Ubiquitin* using the qbase PLUS Data-Analysis Software.

## Supporting Information

Dataset S1 Design of the mRNA-Seq expression profiling experiments, including genotypes, tissues, replicates, total read numbers and mapping rates.
(XLSX)

Dataset S2 Global gene expression profiling from two tissue types, 6^th^ young expanding leaf and TM, grouped as percentages of differentially expressed genes in 12 possible gene action categories when comparing *sp*, *sft/+ sp* and *sft sp*. There are five major classes of gene action: additive (semi-dominant), dominant, recessive, overdominant and underdominant. Subcategories for each major class of gene action are represented by cartoon bar graphs. The first sheet shows the summary statistics of classification in two tissues and results of Fisher's exact tests for significant differences between the percentages in each gene action category. The following sheets show detailed information of the genes, including gene IDs, mean RPKM values, log fold changes for three pairwise comparisons, and P-values from differential expression tests. The 12 gene expression categories are classified based on a threshold of two-fold change and P-value< = 0.01 between genotypes. All possible modes of gene action were observed in both tissues.
(XLSX)

Dataset S3 Marker genes selected by DDI and used in maturation score estimations all meristem DDI analyses involving the 6^th^ expanding leaf, TM stage, and SYM stage. Included are gene IDs and functional annotations from tomato gene annotation iTAG version 2.3 [76].
(XLSX)

Figure S1 *sft/+* mutant heterozygosity delays precocious axillary shoot termination in determinate tomato. (A) Compared to *sp* mutants, *sft/+ sp* plants show delayed primary flowering time on both basal and proximal axillary shoots similar to the main shoot (Figure 2A). Although no statistically significant (P = 0.11), there is a trend towards a delay on the proximal lateral shoots of *sft/+ sp* plants (B) *sft/+ sp* plants produce more sympodial units before sympodial cycling terminates on both basal and proximal axillary shoots, similar to the main shoot (Figure 1B). (C) On both axillary shoots, *sft/+ sp* plants produce more leaves in the first three sympodial units, indicating a delay in precocious termination similar to the main shoot (Figure 1C). (D) Compared to *sp* mutants, *sft/+ sp* plants produce more inflorescences on each plant. Genotypes and sample sizes are shown below, and error bars indicate standard deviations of averages. Statistical significance was tested by Wilcoxon rank sum test, and significance levels are indicated by asterisks (*P<0.05; **P<0.01; ***P<0.001).
(TIF)

Figure S2 Artificial microRNAs (amiRNA) targeting the *SFT* and *FT* genes. (A) Quantitative RT-PCR measurements of tomato *SFT* transcript levels using primers targeting 3′ to the amiRNA binding site. Note that transcript levels show little or no reduction compared to 5′ of the amiRNA binding site (Figure 5B), consistent with reports of primer-dependent transitivity occurring at the 3′ to 5′ direction upon the initial target cleavage, resulting in degradation of the 5′ cleaved product of the target but not the 3′ product [80], [81]. Bars indicate relative expression level and error bars indicate standard deviation among replicates. (B) The *At* pre-amiR-SFT/FT *^At^* ^164b^ and pre-amiR-SFT/FT *^At^* ^319a^ sequences that were introduced into the plants along with theoretical representations of the RNA secondary structure. The fold-back structure in each of the sequences is emboldened and the miRNA sequence is highlighted. (C) 43-day old, long day (18 hours daylight, six hours night) grown *Arabidopsis thaliana* (Landsberg *erecta*) demonstrating the phenotypic effect of *amiR-SFT/FT ^At^* ^164b^ and *amiR-SFT/FT^At^* ^319a^ on *FT* activity and flowering. *35S:amiR-SFT/FT^At^* ^164b^ and *35S:amiR-SFT/FT^At^* ^319a^ transformants exhibit delayed flowering equivalent to *ft* mutant plants.
(TIF)

Figure S3 Dose-dependent suppression of *Arabidopsis thaliana tfl1* mutant flowering time and yield-associated traits when either strong or moderate mutant alleles of *ft* are heterozygous. (A–D) Statistic analyses of *Arabidopsis* phenotypes caused by *ft-2/+* heterozygosity in the *tfl1-2* mutant background. Bars indicate average values with standard deviation. Genotypes and sample size are shown below. Statistical significance was tested by Wilcoxon rank sum test, and significance levels are indicated by asterisks (*P<0.05; **P<0.01; ***P<0.001). (A) Total number of rosette leaves; (B) Total number of axillary shoots; (C) Total number of siliques; (D) Total number of floral buds; Note that number of rosette leaves and siliques showed semi-dominance caused by *ft/+* heterozygosity. (E) Representative plants from left to right of wild type Ler-0 (WT), *tfl1-2* single mutants, *ft-1/+ tfl1-2*, and *ft-1* single mutants. Like for *ft-2*, *ft-1* mutants are completely epistatic over *tfl1-2* mutants, and therefore *ft tfl* double mutants (not shown) are not significantly different from *ft* single mutants (Figure 6).
(TIF)
