# Supplementary figures and images for: Tomato Yield Heterosis Is Triggered by a Dosage Sensitivity of the Florigen Pathway That Fine-Tunes Shoot Architecture
Source: PLoS Genet. 2013 Dec 26;9(12):e1004043. doi: 10.1371/journal.pgen.1004043 (PMC3873276; doi:10.1371/journal.pgen.1004043)

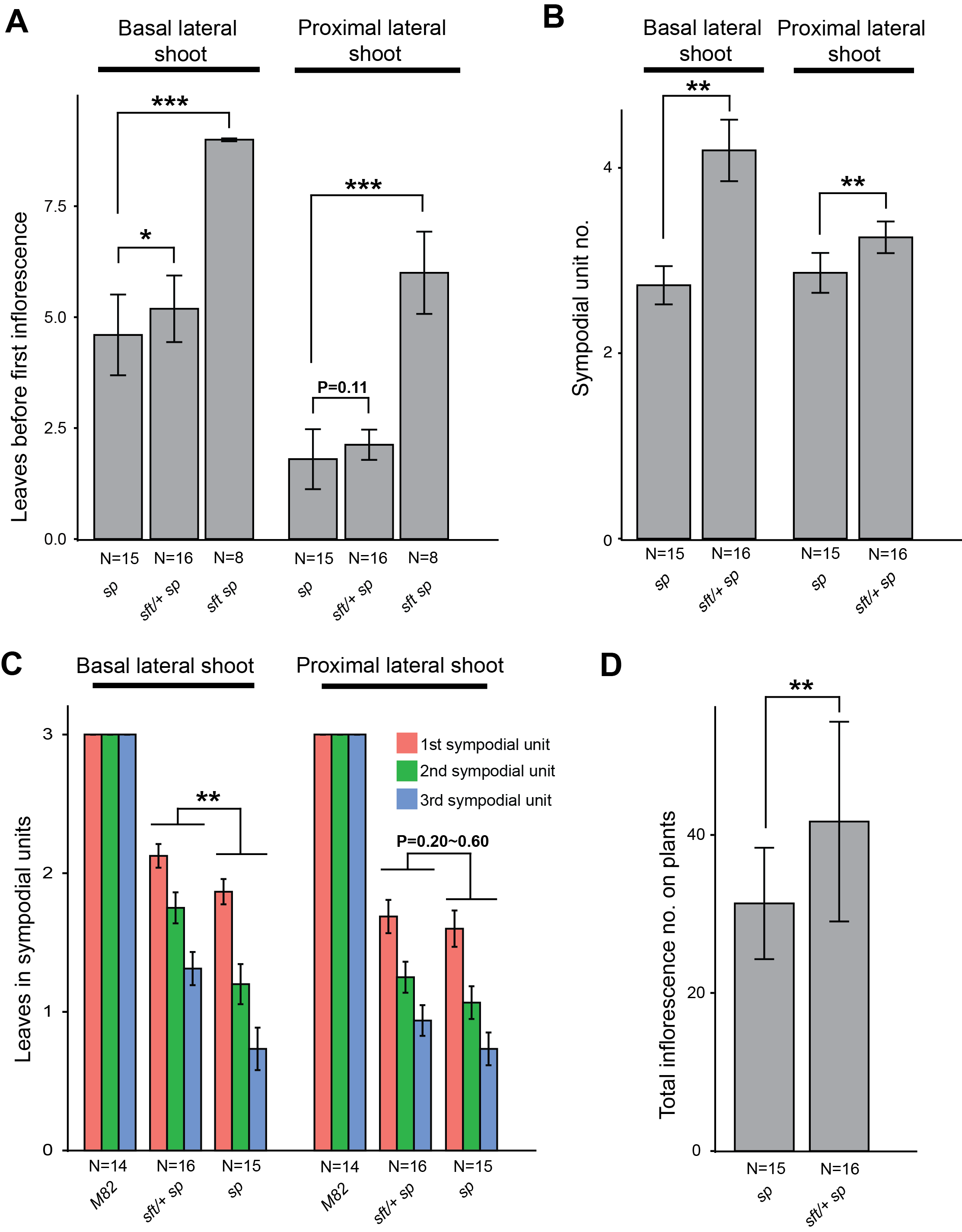

Supplement: Figure S1 — sft/+ mutant heterozygosity delays precocious axillary shoot termination in determinate tomato. (A) Compared to sp mutants, sft/+ sp plants show delayed primary flowering time on both basal and proximal axillary shoots similar to the main shoot (Figure 2A). Although no statistically significant (P = 0.11), there is a trend towards a delay on the proximal lateral shoots of sft/+ sp plants (B) sft/+ sp plants produce more sympodial units before sympodial cycling terminates on both basal and proximal axillary shoots, similar to the main shoot (Figure 1B). (C) On both axillary shoots, sft/+ sp plants produce more leaves in the first three sympodial units, indicating a delay in precocious termination similar to the main shoot (Figure 1C). (D) Compared to sp mutants, sft/+ sp plants produce more inflorescences on each plant. Genotypes and sample sizes are shown below, and error bars indicate standard deviations of averages. Statistical significance was tested by Wilcoxon rank sum test, and significance levels are indicated by asterisks (*P<0.05; **P<0.01; ***P<0.001). (TIF) [file pgen.1004043.s004.tif]

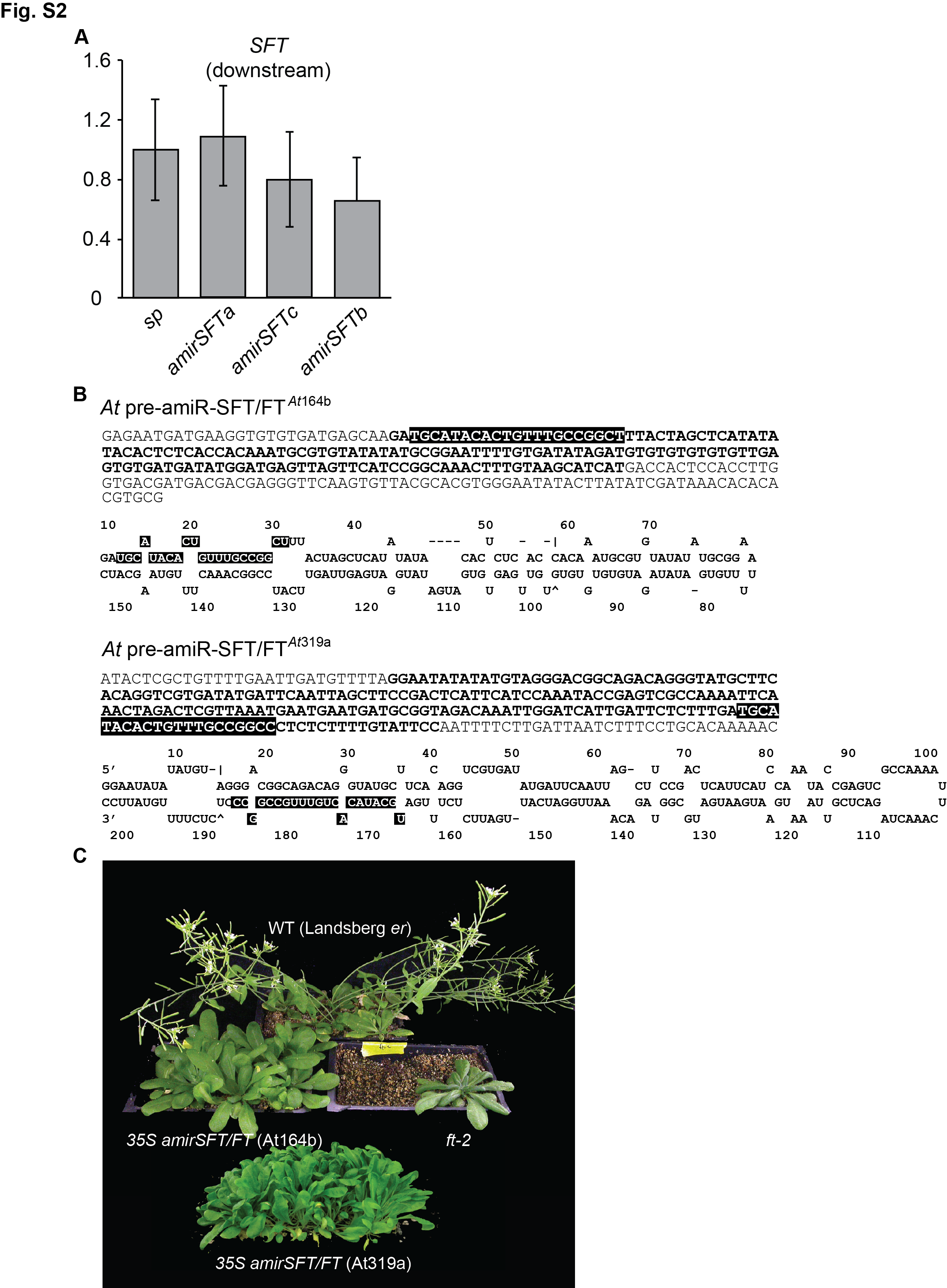

Supplement: Figure S2 — Artificial microRNAs (amiRNA) targeting the SFT and FT genes. (A) Quantitative RT-PCR measurements of tomato SFT transcript levels using primers targeting 3′ to the amiRNA binding site. Note that transcript levels show little or no reduction compared to 5′ of the amiRNA binding site (Figure 5B), consistent with reports of primer-dependent transitivity occurring at the 3′ to 5′ direction upon the initial target cleavage, resulting in degradation of the 5′ cleaved product of the target but not the 3′ product [80], [81]. Bars indicate relative expression level and error bars indicate standard deviation among replicates. (B) The At pre-amiR-SFT/FT At 164b and pre-amiR-SFT/FT At 319a sequences that were introduced into the plants along with theoretical representations of the RNA secondary structure. The fold-back structure in each of the sequences is emboldened and the miRNA sequence is highlighted. (C) 43-day old, long day (18 hours daylight, six hours night) grown Arabidopsis thaliana (Landsberg erecta) demonstrating the phenotypic effect of amiR-SFT/FT At 164b and amiR-SFT/FTAt 319a on FT activity and flowering. 35S:amiR-SFT/FTAt 164b and 35S:amiR-SFT/FTAt 319a transformants exhibit delayed flowering equivalent to ft mutant plants. (TIF) [file pgen.1004043.s005.tif]

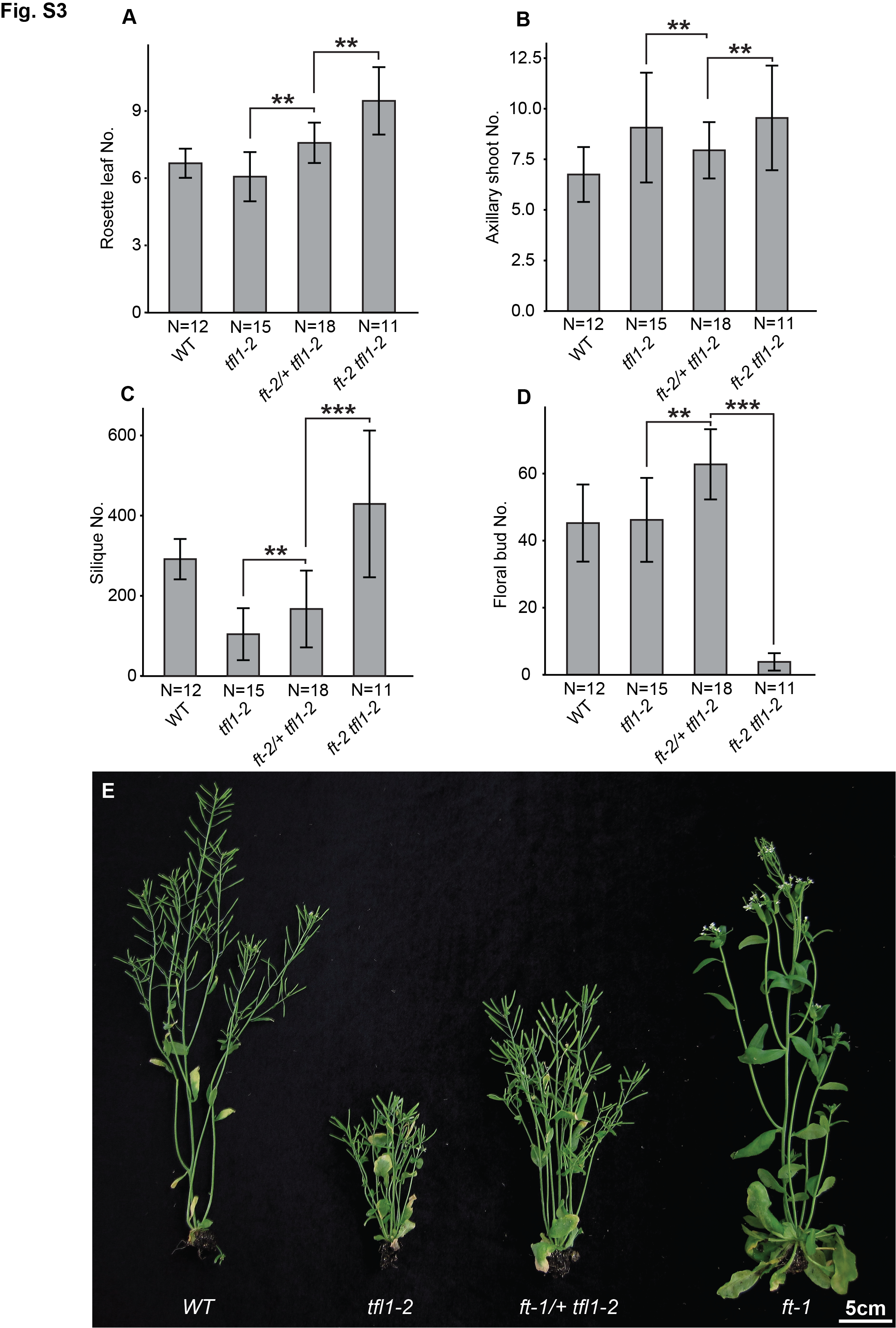

Supplement: Figure S3 — Dose-dependent suppression of Arabidopsis thaliana tfl1 mutant flowering time and yield-associated traits when either strong or moderate mutant alleles of ft are heterozygous. (A–D) Statistic analyses of Arabidopsis phenotypes caused by ft-2/+ heterozygosity in the tfl1-2 mutant background. Bars indicate average values with standard deviation. Genotypes and sample size are shown below. Statistical significance was tested by Wilcoxon rank sum test, and significance levels are indicated by asterisks (*P<0.05; **P<0.01; ***P<0.001). (A) Total number of rosette leaves; (B) Total number of axillary shoots; (C) Total number of siliques; (D) Total number of floral buds; Note that number of rosette leaves and siliques showed semi-dominance caused by ft/+ heterozygosity. (E) Representative plants from left to right of wild type Ler-0 (WT), tfl1-2 single mutants, ft-1/+ tfl1-2, and ft-1 single mutants. Like for ft-2, ft-1 mutants are completely epistatic over tfl1-2 mutants, and therefore ft tfl double mutants (not shown) are not significantly different from ft single mutants (Figure 6). (TIF) [file pgen.1004043.s006.tif]
